# Early identification of women at risk of postpartum depression using the Edinburgh Postnatal Depression Scale (EPDS) in a sample of Lebanese women

**DOI:** 10.1186/s12888-014-0242-7

**Published:** 2014-09-07

**Authors:** Charline El-Hachem, Jihane Rohayem, Rami Bou Khalil, Sami Richa, Assaad Kesrouani, Rima Gemayel, Norma Aouad, Najat Hatab, Eliane Zaccak, Nancy Yaghi, Salimé Salameh, Elie Attieh

**Affiliations:** Department of psychiatry, Hotel Dieu de France- Saint Joseph University, Beirut, Lebanon; Department of obstetrics and gynecology, Hotel Dieu de France- Saint Joseph University, Beirut, Lebanon; Research Unit, department of Nursing, Hotel Dieu de France, Beirut, Lebanon; Saint Joseph University, Beirut, Lebanon

**Keywords:** Depression, Mood disorders, Maternal-child, Pregnancy and postpartum, Measurements/psychometrics

## Abstract

**Background:**

During the postpartum period, women are vulnerable to depression affecting about 10 to 20% of mothers during the first year after delivery. However, only 50% of women with prominent symptoms are diagnosed with postpartum depression (PPD). The Edinburgh Postnatal Depression Scale (EPDS) is the most widely used screening instrument for PPD . The main objectives of this study are to assess whether an EPDS score of 9 or more on day 2 (D2) postpartum is predictive of a depressive episode between days 30 and 40 postpartum (D30-40), to determine the risk factors as well as the prevalence of PPD in a sample of Lebanese women and to determine a threshold score of EPDS predictive of PPD.

**Methods:**

A sample of 228 women were administered the EPDS on D2. An assessment for PPD was done on D30-40 during a telephone interview.

**Results:**

On D2, the average score on EPDS was 7.1 (SD = 5.2) and 33.3% of women had an EPDS score ≥ 9. On D30-40 postpartum, the average score was 6.5 (SD = 4.7) and 19 women (12.8%) presented with PPD. A positive correlation was shown between scores on EPDS on D2 and D30-40 (r = 0.5091, p < 0.0001). A stepwise regression shows that an EPDS score ≥9 on D2 (p < 0.001) and a personal history of depression (p = 0.008) are significantly associated with the diagnosis of PPD on D30-40.

**Conclusion:**

The EPDS may be considered as a reliable screening tool on as early as D2 after delivery. Women with EPDS score ≥ 9 and/or a positive personal history of major depressive disorder should benefit from a closer follow-up during the rest of the post-partum period.

## Background

According to the World Health Organization (WHO), depressive disorders are ranked fourth regarding their global burden of disease and are expected to be ranked second by 2020 [1]. During the postpartum period, women are vulnerable to depression which currently affects about 10 to 20% of mothers in the first year after delivery. However, only 50% of women with prominent symptoms are diagnosed with postpartum depression (PPD) [2]. In the absence of treatment, PPD can lead to chronic depression, disturbances in the interaction between the mother and her newborn and suicide as well as infanticide in rare cases [3]. Accordingly, the need for new screening strategies to detect new cases is very important. According to the Diagnostic and Statistical Manual for Mental Disorders (DSM-IV-TR) and to the International Classification of Diseases (ICD-10), the postpartum period extends to 4 weeks and 6 weeks after birth, respectively. Most studies extend it to one year after delivery [4,5]. Moreover, as per the National Institute for Health and Care Excellence (NICE) guidelines, screening for PPD should be done between 4 to 6 weeks and 3 to 4 months after delivery. The Scottish Intercollegiate Guidelines Network (SIGN) for the detection and management of PPD recommends an initial assessment between 6 and 8 weeks and another one at 3 months. The prevalence of PPD in Asian countries ranges between 3.5% and 63.3% [6]. However, most of the scientific communities’ recommendations regarding the timing for screening and rescreening are not evidence based but based on experts opinion in these groups.

In a Lebanese study done by Chaaya et al. in 2002, the prevalence of PPD in 396 Lebanese women delivering in Beirut and the Bekaa Valley (a rural region in Lebanon) was considered to be 21.3% according to an assessment that has been conducted on the fourth or the fifth months of delivery [7]. All women could be affected regardless of their age, parity, race and socio-economic class [8]. In general, demographic factors do not significantly influence the risk of PPD although some of these variables can be considered as weak risk factors such as unwanted or unplanned pregnancy, low socio-economic status and being single [8]. On the other hand, psychosocial variables such as dissatisfaction within the couple and/or lack of social support are strong predictors of PPD [8,9]. Furthermore, women with the highest risk of PPD are those with a previous history of puerperal psychosis, those with a history of PPD and those who suffered from depressive symptoms during pregnancy [8,10–12].

The Edinburgh Postnatal Depression Scale (EPDS) is the most widely used screening instrument for PPD [13,14]. It is a self-administered questionnaire with 10 items, translated and validated in French and Arabic [15,16]. The questionnaire can be completed in 5 minutes [15,16]. With a threshold of 9, the EPDS has a satisfactory power to detect women complaining of a depressive episode between 4 and 8 weeks postpartum and is therefore considered a good tool for screening of PPD in community settings [17–19].

To our knowledge no studies assessing the reliability of EPDS as an early screening test for PPD exist in a Lebanese sample of women. Thus, the main objectives of this study are:

- To assess whether an EPDS score equal or superior (≥) to 9 on day 2 postpartum (D2) is predictive of a depressive episode between days 30 and 40 postpartum (D30-40).
- To identify risk factors associated with the diagnosis of PPD on D30-40.
- To determine a predictive threshold score of EPDS for the diagnosis of PPD among Lebanese women.
- To determine the prevalence of PPD in a selected sample of Lebanese women.

This is a longitudinal study where participants were assessed twice: on the second day (D2) postpartum and after 30 to 40 days postpartum (D30-40).

## Methods

### Population

This study included women who gave birth between the first of December 2012 and the 31st of March 2013 at Hôtel-Dieu de France Hospital, a university hospital affiliated to Saint-Joseph University in Beirut, Lebanon. The study protocol was approved by Hôtel-Dieu de France hospital’s committee of ethics.

### Scales and questionnaires

The scale used in this study is the EPDS in Arabic, English or French depending on the patient’s choice. A copy of the scale was distributed to women on D2 postpartum. In addition, participants were asked to answer a questionnaire that assessed the following sociodemographic parameters: age, address, nationality, level of education, occupation, personal and family history of depression, perception of the course of pregnancy and delivery, maternal pathology, transfer of the mother or the child to the intensive care unit (ICU), child’s condition at birth, breastfeeding, and fetal medical problems.

### Assessment methods

Most of the women admitted to Hôtel-Dieu de France are usually discharged on the second or the third day after delivery. In order for us to achieve the highest participation rate, women were asked to complete the EPDS and the questionnaire that assesses their sociodemographic parameters on D2 postpartum. Participants with a score superior or equal to 9 were evaluated before discharge from the hospital but were not treated. As a matter of fact, treatment of women with early depressive symptoms is not recommended due to the fact that these symptoms may correspond to baby blues that are prone to remit within 2 weeks after delivery especially when these symptoms are not associated with psychotic symptoms, suicidal plans or attempts [20]. In order to give women with EPDS score ≥ 9 the possibility of getting an adequate management, they were given instructions to contact the maternity department at the hospital or their gynecologist in case their symptoms persisted beyond the two weeks period or aggravated within this period.

In Lebanon, as recommended by the WHO, women are usually asked to consult their gynecologist between 6 and 8 weeks after delivery [21]. We decided to contact them on D30-40 so we can evaluate them before 8 weeks [20]. Therefore, all women with an EPDS score ≥ 9 were contacted by phone on D30-40 to complete the EPDS and a semi-standardized psychiatric interview based on the DSM-IV-TR criteria of a major depressive episode. The telephone interviews were conducted throughout the study by a single psychiatrist (the first author of this manuscript) who knew the results of the first evaluation. Given the difficulty to contact on D30-40 all women included in our sample, and the need to compare women with an EPDS score ≥9 to those with a score < 9 on D2, a control group was randomly selected from the group of women presenting an EPDS < 9. For every woman who scored 9 or more on the EPDS, a woman who scored less than 9 on D2 was randomly selected to undergo an evaluation on D30-40. Women with an EPDS score ≥ 12 and who met criteria for a major depressive episode were considered suffering from a PPD. The threshold of 12 was selected according to the results of many studies in western and eastern countries including Lebanon [2,7,22,23]. If a woman was diagnosed with PPD on D30-40, she has been instructed to report it to her gynecologist on her next visit between 6 and 8 weeks postpartum. In addition, a letter was sent to the gynecologist informing him with the diagnosis and prompting him for psychiatric referral of his patient. All the gynecologists in our study work at the same hospital but had the choice to refer their patients to psychiatrists at our hospital or at other institutions.

### Statistical analysis

Based on EPDS score on D2, the likelihood of developing PPD on D30-40 was calculated using the Odds Ratio (OR) with a confidence interval of 95%. The ability of EPDS on D2 to predict a PPD on D30-40 was evaluated by analyzing the Receiver Operator Characteristic (ROC) curve. The overall accuracy of a tool can therefore be described as the Area Under the Curve (AUC). Setting a cutoff score for the early detection of PPD was carried out through the study of the sensitivity and specificity of the EPDS on D2 to detect a PPD on D30-40. The potential risk factors for postpartum depressive symptoms and PPD were compared between the two groups at both evaluations (D2 and D30-40) using Pearson’s Chi-squared test (corrected by Fisher Exact for small samples). The level of significance was set throughout the study at p ≤ 0.05. A multivariate stepwise regression was performed using all the predictive variables selected in our study. Only the variables with a p value ≤ 0.05 were retained in our final model. Data entry was carried out initially on Epi Info Version 7 and converted to Microsoft Excel. Data analysis was performed using the software Stata/IC version 11.2.

## Results

### Sample characteristics

At the first assessment at D2, 228 women were included in our sample from 245 who were eligible. Refusal to participate and early discharge before D2 were among the reasons behind the lack of participation in the remaining patients. From the first assessment at D2 there were two groups created by their EPDS score, the group of those having an EPDS more or equal to 9 (N = 76) and the group having an EPDS less than 9 (N = 152). At the second assessment, the 76 women who scored ≥9 on the EPDS at D2 were called back, and 71 of them accepted to participate in the assessment. As discussed previously, among the 152 women who scored < 9 on EPDS at D2, we chose 78 women randomly and we called them for a second assessment at D30-40.

Mean age of women included in our sample was 31.7 years (SD = 4.5). 99.3% were Lebanese and the majority (57.9%) lived in Mount-Lebanon (the closest district to Beirut, the capital of Lebanon). 67.4% of women were university graduates, 14.5% had postgraduate education while 25% were unemployed. 8.4% reported a personal history of depression while 7.5% had a positive family history for major depressive disorder. On the other hand, 92.9% of women aknowledged family support especially from their husband, mother or stepmother. 21.7% felt that pregnancy was difficult and 29% found that delivery was hard.

On the other hand, around two thirds of the participants were multiparous (64.4%) and 3.9% gave birth to twins. There were no pregnancies of triplets or quadruplets. 83.4% of women gave birth between 36 and 40 weeks of gestation and 8.3% of babies were born prematurely. More than half of the patients gave birth by Caesarean section (51.1%) while almost a quarter of them underwent a normal vaginal delivery without the use of instruments such as vacuum or forceps (26.2%). In addition, 53.1% of newborns were female and 0.4% of babies were stillborn. Fetal medical problem or obstetrical complications were found in 10.5% of births. In addition, 44.7% of women had a coexisting medical problem, mainly hypertension, preeclampsia, infectious diseases and diabetes. Furthermore, 1.3% of participants were transferred to the intensive care unit (ICU) after delivery. Finally, 58.8% of women chose exclusive breastfeeding while 5.2% opted against it.

### Prevalence of depressive symptoms on D2 postpartum and PPD on D30-40

On D2, the average score on EPDS was 7.1 (SD = 5.2) and 33.3% of women had a score ≥9. On D30-40 postpartum, the average score was 6.5 (SD = 4.7) and 19 women had PPD. Accordingly, the prevalence of PPD has been considered to be 12.8% in our sample.

### Characteristics of women with early depressive symptoms

On D2, the group of women suffering from early depressive symptoms (EPDS ≥9) was compared to the group of asymptomatic participants (Table 1). Among women with an EPDS score ≥9, a higher percentage of women found pregnancy difficult (p < 0.001) or delivery hard (p = 0.002). This group also included a higher incidence of caesarian sections (p = 0.006), adult ICU admissions (p = 0.036), neonatal ICU transfers (18.7% of newborns vs 8.6% in the other group; p = 0.025), as well as a higher percentage of exclusive bottle feeding (p = 0.049).

**Table 1:** **Comparaison at D2 postpartum of the chracteristics of women between the two groups**

		**EPDS <9 (%)**	**EPDS ≥9 (%)**	p*
**N**		152 (66.8%)	76 (33.2%)	
**Mean Age**		31.8	31.2	0.336
**Region of Residency**	**Beirut**	46 (30.3%)	24 (31.6%)	0.621
	**South**	5 (3.3%)	5 (6.6%)	
	**Mount-Lebanon**	92 (60.5%)	40 (52.6%)	
	**North**	3 (2.0%)	3 (3.9%)	
	**Bekaa**	5 (3.3%)	3 (3.9%)	
	**Nabatiyeh**	1 (0.6%)	1 (1.3%)	
**Lebanese**	**Yes**	151 (99.3%)	73 (96.1%)	0.109
	**No**	1 (0.7%)	3 (3.9%)	
**Education**	**Elementary**	1 (0.7%)	1 (1.3%)	0.444
	**Intermediate**	7 (4.6%)	6 (7.9%)	
	**Secondary**	16 (10.6%)	6 (7.9%)	
	**University**	98 (64.9%)	55 (72.4%)	
	**Postuniversity**	26 (17.2%)	7 (9.2%)	
	**Other**	3 (2.0%)	1 (1.3%)	
**Occupation**	**Unemployed**	42 (27.8%)	18 (24.3%)	0.682
	**Employee**	72 (47.7%)	39 (52.7%)	
	**Executive**	12 (7.9%)	8 (10.8%)	
	**Liberal profession**	25 (16.6%)	9 (12.2%)	
**Family Support**	**Absent**	10 (6.6%)	6 (7.9%)	0.785
	**Present**	142 (93.4%)	70 (92.1%)	
**Personnal History of Depression**	**Yes**	10 (6.7%)	8 (10.7%)	0.147
	**No**	140 (93.3%)	67 (89.3%)	
**Family History of Depression**	**Yes**	8 (5.3%)	9 (11.8%)	0.108
	**No**	142 (94.7%)	67 (88.2%)	
**Perception of Pregnancy**	**Easy**	76 (50.7%)	23 (30.3%)	<0.001
	**Average**	53 (35.3%)	25 (32.9%)	
	**Difficult**	21 (14.0%)	28 (36.8%)	
**Perception of Delivery**	**Easy**	62 (41.3%)	21 (28.4%)	0.002
	**Average**	56 (37.3%)	20 (27%)	
	**Difficult**	32 (21.4%)	33 (44.6%)	
**Newborn’s gender**	**Male**	65 (42.8%)	42 (55.3%)	0.091
	**Female**	87 (57.2%)	34 (44.7%)	
**Pregnancy Term**	**<36 WG**	11 (7.2%)	8 (10.7%)	0.390
	**36-40 WG**	130 (85.6%)	59 (78.6%)	
	**>40 WG**	11 (7.2%)	8 (10.7%)	
**Delivery route**	**Vaginal**	48 (31.6%)	12 (15.8%)	0.006
	**Vaccum**	32 (21.1%)	12 (15.8%)	
	**Forceps**	6 (3.9%)	1 (1.3%)	
	**Vaccum + Forceps**	0 (0%)	1 (1.3%)	
	**C-section**	66 (43.4%)	50 (65.8%)	
**Birth order**	**Primiparous**	56 (36.8%)	25 (32.9%)	0.660
	**Multiparous**	96 (63.1%)	51 (67.1%)	
**Twin Pregnancy**	**Yes**	3 (2.0%)	6 (7.9%)	0.063
	**No**	149 (98.0%)	70 (92.1%)	
**Chronic medical problem for the mother**	**Yes**	60 (39.5%)	40 (52.6%)	0.066
	**No**	92 (60.5%)	36 (47.4%)	
**Transfer of the mother to ICU**	**Yes**	0 (0%)	3 (3.9%)	0.036
	**No**	152 (100%)	73 (76.9%)	
**Newborn at birth**	**Dead**	0 (0%)	1 (1.3%)	0.332
	**Alive**	152 (100%)	75 (98.7%)	
**Fœtal medical problem**	**Yes**	12 (7.9%)	12 (15.8%)	0.071
	**No**	140 (92.1%)	64 (84.2%)	
**Newborn transfer to ICU**	**Yes**	13 (8.6%)	14 (18.7%)	0.025
	**No**	139 (91.4%)	61 (81.3%)	
**Breastfeeding**	**No (baby dead)**	0 (0%)	1 (1.3%)	0.049
	**Breastfeeding + 1–2 cups during hospital stay**	33 (21.7%)	10 (13.3%)	
	**No breastfeeding**	4 (2.6%)	7 (9.4%)	
	**Breastfeeding + >2 cups/day**	24 (15.8%)	15 (20%)	
	**Exclusive**	91 (59.9%)	42 (56%)	

WG: Weeks of Gestation; ICU: Intensive Care Unit; *Significance threshold *p* ≤ 0.05.

### Prevalence of PPD on D30-40 depending on the EPDS score on D2 and correlation between the scores on D2 and D30-40

Table 2 shows a significant difference in the prevalence of PPD at D30-40 between the group of women with EPDS score ≥9 and the control group (p = 0.006). 16% of the women who scored 9 or more on the EPDS on D2 suffered from PPD on D30-40. Those women were 1.25 times more likely to develop PPD (95% CI 1.13-1.38). A positive correlation was noted between EPDS scores on D2 and D30-40 (r = 0.5091, p < 0.0001).

**Table 2:** **Comparison of the prevalence of PPD at D30-40 between the two groups**

	**EPDS score at D2**			
	**<9**	**≥9**	***p*** *	**OR [IC 95%]**
N	78	71		
PPD at D30-40	4 (5.1%)	15 (21.1%)	0.006	1.25 [1.13-1.38]

*Significance threshold *p* ≤ 0.05.

### Characteristics of women suffering from PPD

On D30-40, there was a higher percentage of family history of depression (p = 0.001), maternal diseases (p = 0.003), transfers to adult ICU (p = 0.043) and transfers to neonatal ICU (p = 0.05) among the women who suffered from PPD in comparison with those who did not (Table 3).

**Table 3:** **Comparaison of the chracteristics of women both groups at D30-40**

		**Absence of PPD n (%)**	**Presence of PPD n (%)**	p*
**N**		130 (87.2%)	19 (12.8%)	
**Mean Age**		31.7	32.6	0.477
**Region of Residency**	**Beirut**	40 (30.8%)	4 (21.0%)	0.653
	**South**	6 (4.6%)	1 (5.3%)	
	**Mount-Lebanon**	75 (57.7%)	12 (63.1%)	
	**North**	3 (2.3%)	1 (5.3%)	
	**Bekaa**	4 (3.1%)	1 (5.3%)	
	**Nabatiyeh**	2 (1.5%)	0 (0%)	
**Lebanese**	**Yes**	128 (98.5%)	19 (100%)	1.000
	**No**	2 (1.5%)	0 (0%)	
**Education**	**Elementary**	0 (0%)	1 (5.3%)	0.075
	**Intermediate**	10 (7.7%)	0 (0%)	
	**Secondary**	7 (5.4%)	3 (15.8%)	
	**University**	90 (69.2%)	11 (57.9%)	
	**Postuniversity**	19 (14.6%)	4 (21%)	
	**Other**	4 (3.1%)	0 (0%)	
**Occupation**	**Unemployed**	32 (25%)	5 (26.3%)	0.470
	**Employee**	64 (50%)	8 (42.1%)	
	**Executive**	12 (9.4%)	4 (21.1%)	
	**Liberal profession**	20 (15.6%)	2 (10.5%)	
**Family Support**	**Absent**	7 (5.4%)	4 (21.1%)	0.060
	**Present**	123 (94.6%)	15 (78.9%)	
**Personnal History of Depression**	**Yes**	9 (7%)	4 (21.1%)	0.066
	**No**	120 (93%)	15 (78.9%)	
**Family History of Depression**	**Yes**	9 (7%)	7 (36.8%)	0.001
	**No**	120 (93%)	12 (63.2%)	
**Perception of Pregnancy**	**Easy**	54 (41.9%)	4 (21.1%)	0.130
	**Average**	44 (34.1%)	7 (36.8%)	
	**Difficult**	31 (24%)	8 (42.1%)	
**Perception of Delivery**	**Easy**	44 (34.4%)	4 (22.2%)	0.544
	**Average**	41 (32%)	6 (33.4%)	
	**Difficult**	43 (33.6%)	8 (44.4%)	
**Newborn’s gender**	**Male**	63 (48.5%)	12 (63.2%)	0.326
	**Female**	67 (51.5%)	7 (36.8%)	
**Pregnancy Term**	**<36 WG**	10 (7.8%)	3 (15.8%)	0.473
	**36-40 WG**	109 (84.4%)	15 (78.9%)	
	**>40 WG**	10 (7.8%)	1 (5.3%)	
**Delivery route**	**Vaginal**	27 (20.8%)	3 (15.8%)	0.553
	**Vaccum**	30 (23.1%)	2 (10.5%)	
	**Forceps**	2 (1.5%)	0 (0%)	
	**Vaccum + Forceps**	1 (0.8%)	0 (0%)	
	**C-section**	70 (53.8%)	14 (73.7%)	
**Birth order**	**Primiparous**	50 (38.5%)	5 (26.3%)	0.446
	**Multiparous**	80 (61.5%)	14 (73.7%)	
**Twin Pregnancy**	**Yes**	6 (4.6%)	0 (0%)	1.000
	**No**	124 (95.4%)	19 (100%)	
**Chronic medical problem for the mother**	**Yes**	54 (41.5%)	15 (78.9%)	0.003
	**No**	76 (58.5%)	4 (21.1%)	
**Transfer of the mother to ICU**	**Yes**	1 (0.8%)	2 (10.5%)	0.043
	**No**	129 (99.2%)	17 (89.5%)	
**Newborn at birth**	**Dead**	1 (0.8%)	0 (0%)	1.000
	**Alive**	129 (99.2%)	19 (100%)	
**Fœtal medical problem**	**Yes**	15 (11.5%)	3 (15.8%)	0.704
	**No**	115 (88.5%)	16 (84.2%)	
**Newborn transfer to ICU**	**Yes**	15 (11.6%)	5 (26.3%)	0.050
	**No**	114 (88.4%)	14 (73.7%)	
**Breastfeeding**	**No (baby dead)**	1 (0.8%)	0 (0%)	0.272
	**Breastfeeding + 1–2 cups during hospital stay**	21 (16.2%)	1 (5.3%)	
	**No breastfeeding**	6 (4.6%)	3 (15.8%)	
	**Breastfeeding + >2 cups/day**	22 (16.9%)	2 (10.5%)	
	**Exclusive**	80 (61.5%)	13 (68.4%)	

WG: Weeks of Gestation; ICU: Intensive Care Unit; *Significance threshold *p* ≤ 0.05

### Multivariate Stepwise regression to identify significant factors associated with PPD

A stepwise regression shows that an EPDS score ≥9 on D2 (p < 0.001) and a personal history of depression (p = 0.008) are significantly associated with the diagnosis of PPD on D30-40 (Table 4).

**Table 4:** **Significant factors associated to PPD according to the stepwise analysis**

**Risk Factor**	**OR (95% CI)**	***p*** **-value**
EPDS score ≥9 on D2	1.23 (1.11′-1.36)	<0.001
Personal history of depression	6.80 (1.65-28.04)	0.008

### Sensitivity and specificity of the EPDS for detecting a PPD on D30-40

Sensitivity is the proportion of women diagnosed with PPD on D30-40 based on the EPDS score and the semi-standardized interview, that were found to have depressive symptoms on D2 according to their EPDS score. Specificity is the proportion of women exempt from PPD on D30-40, that were not found to have depressive symptoms on D2. Based on the ROC analysis, a score of 7 on the EPDS had the highest sensitivity and specificity combined (89.5% and 47.7%, respectively), and was therefore selected as threshold score for the detection of PPD (Table 5). Moreover, the area under the ROC curve is 0.8243 with a 95% confidence interval of [0.72354-0.92505] that does not include 0.5. So the EPDS score ≥9 at D2 can significantly detect mothers at risk of PPD at D30-40.

**Table 5:** **Results of the ROC analysis of EPDS at D2 post-partum, concerning the diagnosis of PPD according to DSM IV criteria**

**Cut-Off score**	**Sensitivity (%)**	**Specificity (%)**
5	100	33.08
6	94.74	40.00
**7**	**89.47**	**47.69**
8	78.95	50.00
9	78.95	56.92
10	78.95	70.00
11	78.95	77.69
12	73.68	82.31

According to its sensitivity and specificity, a score of 7 on the EPDS could be selected as a threshold score for the detection of PPD.

## Discussion

In this study, in concordance with many other studies [7,8,22–31], no association was shown between PPD and any of the following variables: age of the mother, gender of the child, route of delivery, perception of pregnancy, perception of delivery, parity and maturity at childbirth. Despite the fact that the majority of women (81.9%) were university graduates, there was no association between the level of education and PPD. This finding converges with that found in a Turkish study of 318 women at 6 weeks postpartum [26]. Conversely, other studies showed that a low level of education was more frequently observed among postpartum depressed women [7,32]. For example, according to Chaaya et al., PPD was associated with a lower level of education in a subgroup of women living in a rural region (Bekaa Valley), but no relation was found among women living in Beirut [7].

Most women in our sample reside in Beirut and Mount Lebanon that are considered two highly urbanized areas. In our study, the risk of depressive episodes at 4–6 weeks postpartum was not increased in unemployed women contrarily to the findings of other studies where PPD was more common among housewives [7,26,33]. While resuming a social and productive life may seem protective against the development of PPD, it may as well be percieved by the woman as an additional burden. Moreover, the lack of family support was almost significantly correlated to the onset of depressive symptoms in our study (p = 0.06). Concordantly, Chaaya et al. as well as many other authors observed an increase in the likelihood of scoring high on the EPDS score and of PPD with the lack of family and social support [7,22,25,34,35]. The fact that the lack of family support, as a risk factor of PPD, tended to be significant while being found overtly significant in other studies could be partly due to the fact that women in our study were inquired regarding this factor, on D2, before they truly needed the support.

As in many other studies, a positive personal history of depression has been confirmed to be a risk factor for PPD in our study [7,22,26,33]. In addition, we found a greater percentage of women with a positive family history of depression among mothers with PPD on D30-40. This finding is consistent with other studies showing a significant association between PPD and a positive family history of depression [7,36]. Moreover, an increase in neonatal ICU admissions was significantly found in the group of women with an EPDS score ≥9 (p = 0.05). Similarly, in a study including 103 Croatian women with no known psychiatric history, maternal diseases or maternal complications at birth, the presence of a fetal medical problem was more frequent among women with acute depressive symptoms three days after childbirth [37]. Our findings match those of the latter study with a significantly higher proportion of maternal and newborn ICU transfers in women with early depressive symptoms (p = 0.036 and p = 0.025 respectively) and/or EPDS score ≥9 on D30-40 (p = 0.043 and 0.05 respectively). In our sample as well as in that of Chaaya et al., the presence of a maternal chronic medical problem whether related or not to pregnancy, negatively affected the woman’s mood at 4–6 weeks [7]. Finally, women who opt more for breastfeeding instead of artificial feeding techniques were found, in our sample, to be less prone to develop PPD. This is in concordance with the findings of Quelopana et al. where depressed women have been found less prone to initiate breastfeeding [35].

In our study, the prevalence of PPD on D30-40 is around 12.8% which is considered close to the prevalence found in the sample of the study done by Chaaya et al. selected from an urban area (16%) but lower than that found in the rural area (26%) [7]. Women from our sample with a score ≥9 on D2 postpartum had a 25% higher risk of developing PPD on D30-40 (1.25 times higher risk). A higher risk of PPD was found by Dennis et al. when the first assessment with EPDS done at one week postpartum presented a score > 9 [19]. The risk of PPD at the second assessment done at the fourth week and the third assessment done at the eighth week was clearly higher (30.3 and 19.1 times higher respectively) [19]. However, in the latter study, the diagnosis of a depressive episode between 4 and 8 weeks postpartum relied solely on a score of 12 or above on the EPDS with no conducted interviews to confirm the diagnosis [19]. Therefore, despite the fact that an EPDS score ≥9 on D2 weakly predicts a PPD on D30-40, it allows us to select the patients who need a closer follow-up during the post-partum period. Yet, the limitation in this screening method will remain the high number of monitored women who will not develop PPD. Nevertheless, most of these women were pleased by the important support from the hospital’s team and did not object to being interviewed again on D30-40 even though they did not develop PPD.

The multivariate stepwise regression showed that the two factors significantly associated with PPD are an EPDS score ≥ 9 on D2 and a positive personal history of depression. Therefore, we can speculate that women who have any one of those two risk factors are candidates for a close follow-up because of their higher risk for PPD on D30-40. In addition, we found a positive correlation between scores on the EPDS on D2 and D30-40. Accordingly, the intensity of the depressed mood on as early as D2 seems to be predictive of a later risk of PPD (r = 0.51). This result is similar to that found by Teissedre and Chabrol who evaluated women starting the third day postpartum [2]. Dennis also found a significant correlation between the EPDS scores on the first week and those at 4 and 8 weeks (r = 0.72 and 0.65, respectively) [19]. Teissedre and Chabrol suggested that the score should be reduced from 11.5/30 to 9/30 when the EPDS is used in the third postpartum day [2]. However, our findings allow us to suggest an EPDS threshold of 7 on D2 postpartum, as the best cutoff score (most sensitive and specific) predictive of a depressive episode on D30-40 postpartum.

Our sample is smaller than that selected in most of other studies. This fact might explain the non significant correlations found regarding the role of some known risk factors in the occurrence of PPD. In addition, our study included women who gave birth at Hôtel-Dieu de France, a reference hospital that usually treats women from all over the country. However, during the recruitment period, the hospitalized women were mainly residents in two urban regions, Beirut and Mount-Lebanon, which could partly explain the high percentage of university graduates among them. However, with the lack of national studies assessing the prevalence of women with a higher education, we cannot clearly determine if the education level of women in our sample is significantly different from the rest of the population. On the other hand, the high rate of caesarean sections in our sample can be justified by the fact that Hôtel-Dieu de France hospital in Lebanon is considered a tertiary reference center for maternal and fetal medical problems. Our results match the current trend of cesarean sections delivery especially in women selected from urban regions [26,38]. Furthermore, in our study, the diagnosis of PPD was based on a semi- standardized interview along with EPDS. The use of a standardized diagnostic tool would have been more pertinent but given the fact that interviews on D30-40 were conducted over the phone instead of live interviews, completing additional scales would have been a much more difficult task.

## Conclusion

The present study demonstrates that an EPDS ≥ 9 may be considered as a reliable indicator, on as early as D2 after delivery, for a potential risk of developing PPD in a sample of Lebanese women. Women with EPDS score ≥ 9 and/or a positive personal history of major depressive disorder should benefit from a closer follow-up during the rest of the post-partum period.

## Abbreviations

AUC: Area under the curve
D2: Second day postpartum
D30-40: 30 to 40 days postpartum
EPDS: Edinburgh postnatal depression scale
ICU: Intensive care unit
PPD: Post-partum depression
